# RITS: a toolbox for assessing complex interventions via interrupted time series models

**DOI:** 10.1186/s12874-021-01322-w

**Published:** 2021-07-08

**Authors:** Maricela Cruz, Marco A. Pinto-Orellana, Daniel L. Gillen, Hernando C. Ombao

**Affiliations:** 1 Kaiser Permanente Washington Health Research Institute, Seattle, WA, USA; 2 Department of BiostatisticsUniversity of Washington, Seattle, WA, USA; 3 Department of Mechanical, Electronics and Chemical Engineering, Metropolitan Oslo University, Oslo, Norway; 4 Department of StatisticsUniversity of California Irvine, Irvine, California, USA; 5 Computer, Electrical and Mathematical Science and Engineering DivisionKing Abdullah University of Science and Technology, Thuwal, Saudi Arabia

**Keywords:** Change-point detection, Complex interventions, Interrupted time series, Policy interventions, Segmented regression, Toolbox

## Abstract

**Background:**

Various interacting and interdependent components comprise complex interventions. These components create difficulty in assessing the true impact of interventions designed to improve patient-centered outcomes. Interrupted time series (ITS) designs borrow from case-crossover designs and serve as quasi-experimental methodology able to retrospectively assess the impact of an intervention while accounting for temporal correlation. While ITS designs are aptly situated for studying the impacts of large-scale public health policies, existing ITS software implement rigid ITS methodology that often assume the pre- and post-intervention phases are fully differentiated (by a known change-point or set of time points) and do not allow for changes in both the mean functions and correlation structure.

**Results:**

This article describes the Robust Interrupted Time Series (RITS) toolbox, a stand-alone user-friendly application researchers can use to implement flexible ITS models that estimate the lagged effect of an intervention on an outcome, level and trend changes, and post-intervention changes in the correlation structure, for single and multiple ITS. The RITS toolbox incorporates a formal test for the existence of a change in the outcome and estimates a change-point over a set of possible change-points defined by the researcher. In settings with multiple ITS, RITS provides a global over-all units change-point and allows for unit-specific changes in the mean functions and correlation structures.

**Conclusions:**

The RITS toolbox is the first piece of software that allows researchers to use flexible ITS models that test for the existence of a change-point, estimate the change-point (if estimation is desired), and allow for changes in both the mean functions and correlation structures at the change point. RITS does not require any knowledge of a statistical (or otherwise) programming language, is freely available to the community, and may be downloaded and used on a local machine to ensure data protection.

## Background

Evaluating the impact of complex interventions on patient-centered outcomes is a critical concern in public health, as natural experiments are generally not scientifically controlled. Randomized controlled trials, the “gold standard” for evidence-generation of health interventions, are often infeasible and impractical regarding health care reform [1]. As such, data from natural experiments in public health do not typically arise from randomized controlled trials [2]. According to the 2018 Annual Review of Public Health, interrupted time series (ITS) designs are aptly situated for studying the impacts of large-scale public health policies [3]. ITS designs borrow from traditional case-crossover designs and serve as quasi-experimental methodology able to assess the impact of an intervention retrospectively and account for temporal dependency [4].

Natural experiment data regularly present as ITS: sequences of measurements for an outcome (e.g., patient experience scores) recorded at various time points before and after an intervention. Simple comparisons of the mean pre- and post-intervention, say via a t-test, do not provide the statistical rigor needed to account for contextual factors and preexisting trends encountered in ITS data [5]. The most utilized statistical methodology for analyzing ITS data is segmented regression, a powerful methodology accounting for underlying trends, including outcome trajectories and correlation [6–8]. Segmented regression was first introduced in [9], closely followed by [10]. Since then, segmented regression has been used in many forms and disciplines, including health services research, economics, and education.

Traditional segmented regression a priori assumes the change-point to be a specific value, typically at or around the formal intervention, to fully differentiate the pre- and post-intervention phases [11]. The change-point is defined as the time point at which a change occurs in the time series, i.e., the first time point in the post-intervention phase [8]. A change in the time series is not limited to a difference in the mean level. Changes in the time series may be a change in the mean function (e.g., slope), a difference in the variance or spread around the mean function, or a change in the time series data’s autocorrelation structure. An intervention interested in increasing the stability and predictability of an outcome may not lead to a change in the time series mean level but may have decreased outcome variability and increased autocorrelation. Decreasing outcome variability leads to less extreme values while increasing autocorrelation leads to a more predictable outcome. When assessing the impact of an intervention, differences in variability and autocorrelation are therefore important to capture alongside mean differences. Our proposed RITS and R-MITS approaches capture all of the aforementioned time series’ changes.

An instantaneous intervention effect is often assumed, i.e., the change-point is often set to the intervention time. Specification of the change-point as the time of intervention does not, however, represent the reality that complex interventions may have varied effects and take time to manifest change, and can therefore lead to incorrect measures of the intervention’s effect. Prevalent approaches to overcoming this limitation are to remove a specific set of time points from the analysis [7, 11]. This censoring (i.e., removal of time points) not only omits data, but also potentially biases parameter estimates, as the study team decides which time points to remove. Segmented regression methods, furthermore, neglect plausible changes in temporal dependence and variability post-intervention and restrict the analysis to a single unit. These are serious limitations because these methods ignore interpretable changes in the higher-order moments of the response and do not take advantage of all available data that may provide information on the intervention’s effect.

**Fig. 1:**
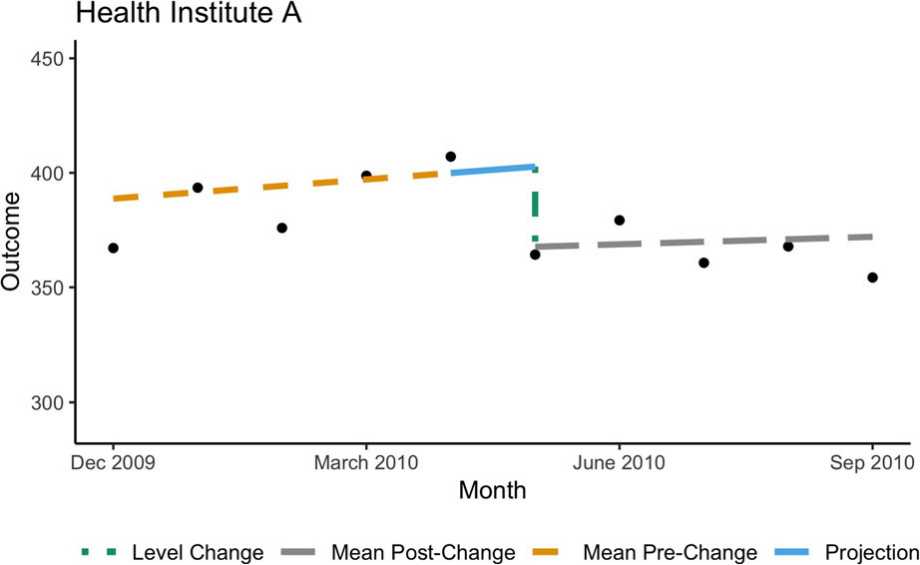
An example of an estimated mean function for sample data. Depicts: (1) the estimated mean function for the pre- and post-change-point phases, (2) the projection of the mean function at the change-point based on the pre-change-point regression, and (3) the change in level

The public health community has adopted two measures to assess the impact of an intervention: level change and trend change. It is necessary to report both level change and trend change when interpreting the results of an ITS study [12]. Level change is interpreted as the discontinuity between the projected mean (based on the pre-change-point phase) and the estimated mean post-change-point, i.e., the anchored intercept at the change-point, depicted graphically in Fig. 1. Trend change quantifies the impact of the intervention on the overall trajectory of the mean function, i.e., the change in slopes post-intervention.

The ‘Robust-ITS’ model proposed in [13] is based on segmented regression and addresses two of the aforementioned limitations by estimating from the data — rather than merely specifying a priori — the change-point and allowing for changes in an outcome’s mean function and correlation structure post-intervention. Robust-ITS provides estimates of the level change and trend change, as well as changes in the adjacent correlation (correlation between two consecutive time points) and variance of the response. Thus, using Robust-ITS allows researchers to estimate (rather than merely assume) the lagged effect of a health care intervention on a health outcome. Moreover, researchers can determine whether health outcomes are more predictable — stronger temporal dependence and smaller variability suggests a more predictable, and thus more desirable outcome — post-intervention.

[14] propose the ‘Robust Multiple ITS’ (R-MITS) model, an extension of Robust-ITS for multi-unit data (e.g., multiple ITS of the same outcome for different units, health systems, clusters, etc.), and the ‘supremum Wald test’ (SWT). The SWT is a formal test used to determine the existence of a change-point over a set of possible change-points specified by the intervention experts. R-MITS borrows information across hospital units to increase efficiency, estimates a global over-all units change-point, and also allows for changes in the mean functions, temporal dependence, and variability of an outcome across units.

This paper focuses on enabling the implementation of Robust-ITS, R-MITS, and the SWT in the Robust Interrupted Time Series (RITS) toolbox, a stand-alone user-friendly, open-source and cross-platform application freely available to the community. The RITS Toolbox has been implemented using web technologies to ensure smooth and uniform execution across platforms. The user interface has been developed in JavaScript language programming, while the statistical model has been independently implemented in Nim language programming to ensure better memory and speed efficiency. The source code is maintained in a public GitHub repository [15]. Updated links for downloading the toolbox are included in the repository.

### Overview of methods

The RITS toolbox implements (i.) Robust-ITS for a single ITS, (ii.) R-MITS for multiple ITS, and (iii.) the SWT to test for the existence of a change-point in the mean function(s) over a set of user-specified plausible change-points. The Robust-ITS and R-MITS models differ in expression solely through their capability to handle single or multiple ITS; we, therefore, follow the notation for R-MITS [14]. Let *N* denote the number of ITS or units. If *N*=1, then RITS implements the Robust-ITS model, otherwise, R-MITS is implemented.

As in [13] and [14], we make a clear distinction between the **time of intervention** and the **change-point**. The time point at which the intervention is formally introduced is denoted by *t*^∗^ and the change-point by *τ*. It may be true that *t*^∗^=*τ*, but this need not always be the case. For example, an intervention learning effect may exist, leading to a delay in the realization of the full intervention impact. Conversely, there could be an anticipatory effect if there is a period between announcement of the intervention and its implementation during which some intervention changes are slowly introduced [16].

Let *y*_*it*_ denote the outcome of interest for unit *i* at time *t*, with $i \in \{1, \dots, N\}, t \in \{1, \dots, n_{i}\},$ and *n*_*i*_ denoting the time series length for unit *i*. Then, the general regression is defined as

1 $$  y_{it} = \mu_{it} + \epsilon_{it},  $$

where *μ*_*it*_ is the mean function and *ε*_*it*_ is the stochastic process that models fluctuations around the mean functions and auto-correlation within the time series. The mean function of the outcome of interest for unit *i* at time *t* is

2 $$  \mu_{it} = \left\{ \begin{array}{ll} \beta_{i0}^{\tau} + \beta_{i1}^{\tau} \, t, & \, t < \tau \\ \left(\beta_{i0}^{\tau} + \delta_{i}^{\tau}\right) \, + \left(\beta_{i1}^{\tau} + \Delta_{i}^{\tau}\right) t, & t \geq \tau \end{array} \right.,  $$

where $\beta _{i0}^{\tau }$ denotes the intercept of the mean function prior to the change-point, $\beta _{i1}^{\tau }$ denotes the slope of the outcome prior to the change-point, $\beta _{i0}^{\tau } + \delta _{i}^{\tau }$ is the intercept of the post-intervention phase, $\beta _{i1}^{\tau } + \Delta _{i}^{\tau }$ is the slope of the post-intervention phase for the outcome in unit *i*, and *τ* denotes the global over-all-unit change-point of the response. In the case with only one unit, *τ* denotes the change-point for that one time series. If $\delta _{i}^{\tau }=\Delta _{i}^{\tau }=0,$ then there is no change in the mean function of unit *i* before and after *τ*.

Recall, the two metrics that are most commonly reported in ITS studies are level change and trend change [12]. Level change is denoted by $\delta _{i}^{\tau } + \Delta _{i}^{\tau } \tau $ and interpreted as the change in anchored intercept (anchored at the change-point); see Fig. 1 for visualization. Trend change is the change in slope and thus denoted by $\Delta _{i}^{\tau }$ in Eq. (2).

The RITS toolbox allows the correlation structure to be modeled with an independent, exchangeable, or AR(1) correlation structure (default). In all cases the correlation structure is assumed to vary pre- and post-change-point. For simplicity we include the model specification for the AR(1) case only, as that is the default and original correlation structure assumed in both the Robust-ITS and R-MITS models. Assuming an AR(1) process to model the correlation structure via the residuals, RITS conditions on the first observation, *y*_*i*1_ for all *i*. Let $r_{it} = y_{it} - \widehat {\mu }_{it}$ denote the residuals, with $i \in \{1, \dots, N\}, t \in \{2, \dots, n_{i}\},$ and $\widehat {\mu }_{it}$ as an estimate of the mean function for unit *i* at time *t*. The residuals are modeled as:

3 $$  {}r_{it} = \left\{ \begin{array}{lrr} \phi_{i1} (\tau) \, r_{i, t-1} + e_{it, 1}, & & \, \, \, \, \, \, \, \, \, \, \, \, \, \, \, \, \, 1 \, < \, t \, \leq \, \widehat{\tau} -1\\ \phi_{i2} (\tau) \, r_{i, t-1} + e_{it, 2} & & \widehat{\tau} -1 \, < \, t \, \leq \, n. \end{array} \right.,  $$

with *ϕ*_*i*1_(*τ*) and *ϕ*_*i*2_(*τ*) in the interval (−1,1) for all *i*, as they are correlations. Specifically, *ϕ*_*i*1_(*τ*) is the correlation between time point *t* and *t*+1 where *t* and *t*+1 belong to the pre-change-point phase and $\phi _{i1}^{\vert h \vert }(\tau)$ is the correlation between two time points *h* units away, say *t* and *t*+*h*, both in the pre-change-point phase. The auto-regressive coefficient of the post-change-point phase, *ϕ*_*i*2_(*τ*), has a similar interpretation. Note, $e_{it, j} \stackrel {iid}{\sim } N \left (0, \sigma _{iw, j}^{2} \right)$ for *i*∈{1,2}, and thus, the variance of the response at any time point *t* is $ \sigma _{it}^{2} = \frac { \sigma _{iw, j}^{2}}{1 - \phi _{ij}(\tau)^{2}}$ for *j*∈{1,2}.

#### Parameter estimation

The mean function parameters are estimated simultaneously with the stochastic component parameters and the change-point. First, the set of potential change-points is established by the application experts. Then, for each possible change-point, the generalized least squares estimates of the mean function parameters are obtained, followed by the method of moments estimates of the stochastic component parameters, via an iteratively re-weighted least squares algorithm (IRLS). For further details about the estimation procedure refer to [13] and [14].

The estimated change-point corresponds to the time point in the set of possible change-points that maximizes the conditional likelihood. The final estimates of the mean function and correlation parameters are obtained by the IRWLS algorithm at the estimated change-point.

#### Formal test for the existence of a change-point

RITS implements the supremum Wald test (SWT), a test for the existence of a change-point in the outcome mean functions [14]. The supremum Wald test calculates a multivariate Wald test statistic for every possible change-point and applies the Benjamini-Hochberg method to adjust for multiplicity. If any of the multivariate Wald statistics provide significant results when compared to the Benjamini-Hochberg corrected critical values, the SWT concludes that a change-point exists for at least one of the units. The SWT results in a binary decision of whether or not a change-point exists in any of the unit-specific mean functions.

The SWT focuses on determining the existence of a change-point across the unit specific mean functions, i.e., for each *q*∈*Q*, where *Q* denotes the set of possible change-points, the SWT tests

$$\begin{aligned} H_{0} : \, \left[\begin{array}{c} \delta_{1} \\ \Delta_{1} \\ \vdots \\ \delta_{N} \\ \Delta_{N} \end{array}\right] = \left[\begin{array}{c} 0 \\ 0 \\ \vdots \\ 0 \\ 0 \end{array}\right] \text{vs.} \,\,\,\,\, H_{a} : \, \ \left[\begin{array}{c} \delta_{1} \\ \Delta_{1} \\ \vdots \\ \delta_{N} \\ \Delta_{N} \end{array}\right] \neq \left[\begin{array}{c} 0 \\ 0 \\ \vdots \\ 0 \\ 0 \end{array}\right]. \end{aligned} $$

Let $\vec {\beta }^{1} \equiv \left [\beta _{1, 0}^{1} \,\,\, \beta _{1, 1}^{1} \,\, \delta _{1} \,\, \Delta _{1} \, \dots \, \beta _{N, 0}^{1} \,\,\, \beta _{N, 1}^{1} \,\, \delta _{N} \,\, \Delta _{N} \right ]^{'}$ be the vector of mean function parameters under the *alternative* hypothesis (with 4×*N* elements), $\vec {\beta }^{0} \equiv \left [\beta _{1, 0}^{0} \,\,\, \beta _{1, 1}^{0} \dots \, \beta _{N, 0}^{0} \,\,\, \beta _{N, 1}^{0} \right ]^{'}$ be the vector of mean function parameter estimates under the *null* hypothesis (with 2×*N* elements), and **C** denote the contrast matrix that selects the *δ*_*i*_ and *Δ*_*i*_ entries for all *i* from $\vec {\beta }^{1}.$ The above hypotheses can then be simply rewritten as

$$H_{0} : \, \mathbf{C} \, \vec{\beta}^{1} = \vec{0} \,\, \, \text{vs.} \,\, \, H_{a} : \, \mathbf{C} \, \vec{\beta}^{1} \neq \vec{0}. $$

The multivariate Wald test statistic is therefore

4 $$ W = \left(\mathbf{C} \widehat{\vec{\beta}}^{1} \, \right)^{'} \, \left[\mathbf{C} \,\, \mathbf{\widehat{V}} (\widehat{\vec{\beta}}^{0}) \, \mathbf{C}^{\prime}\right]^{-1} \left(\mathbf{C} \widehat{\vec{\beta}}^{1} \, \right) \, \overset{H_0}{\overset{.}{\sim}} \, \chi^{2}_{2N},  $$

with $ \mathbf {\widehat {V}}\left (\widehat {\vec {\beta }}^{0}\right)$ denoting the estimator of the variance covariance matrix of $\widehat {\vec {\beta }}^{0},$ the estimator of $\vec {\beta }^{0}.$ Note, $ \mathbf {\widehat {V}}\left (\widehat {\vec {\beta }}^{0}\right)$ is block diagonal (with each block corresponding to one unit) because we assume that the units are independent. The multivariate Wald statistic is calculated for each *q*∈*Q*. If any of the multivariate Wald tests provide significant results when compared to the Benjamini-Hochberg corrected critical values, the SWT concludes that a change-point exists for at least one of the units over the set of possible change-points.

## Implementation

The RITS toolbox is a cross-platform open-source program developed using web technologies to assess the impact of interventions using two statistical models: the Robust-ITS and R-MITS models for single- and multi-unit data. The user interface aims to smoothly interact with data while prioritizing interpretability via a high-level abstraction of the statistical model and internal computational details.

The toolbox is designed with an architecture based on classes and components organized in logical packages grouped into two subsystems: the modeling and the graphical user interface subsystem – each one written in a different language and under distinct sets of dependent libraries.

The user interface is delivered using hybrid web-desktop technologies with integration between user-friendly modern features from web widgets and native operating system (OS) services. The communication with the user’s OS is achieved using the Electron framework. This technical choice enables the program to be executed across platforms consistently without the need to install any additional software. Note that the proper functionality of the main features is evaluated on three major operating systems: MacOS, Windows, and GNU/Linux.

RITS toolbox’s users can manage potentially sensitive information. We, therefore, evaluate and examine the libraries involved in the program to ensure that the framework will operate fully offline. There is full control over the data used inside the software (web-based libraries do not always meet with this requirement).

Compared to the user interfaces, the RITS’ statistical models have a different set of computational requirements, including a higher degree of numerical accuracy, better memory management and time efficiency. In order to meet these requirements, we implement the subsystem in a different programming environment that can support the specifications. The source code is written in the Nim language programming using an object-oriented programming (OOP) paradigm.

The statistical modeling subsystem is written in the Nim programming language with a collection of OS-independent libraries that allow this software component to be embedded as a standalone library in C applications or included in web applications written in JS. Both back-ends are provided by the programming language, which ensures the inter-compatibility of the application with potential extensions. The vast majority of the source code in this subsystem is written as an object-oriented paradigm (OOP) for extensibility and debugging purposes. The OOP style enables the integration of future models in the RITS toolbox. In addition, the chosen programming framework provides a robust type system that encourages the consistency of input data at compile-time without memory and time overhead. The source code written with this language is later compiled into a highly optimized JavaScript (JS) that is later coupled into the user interface. The model’s code is highly optimized to ensure the overall efficiency of the RITS software.

The user interface (UI) subsystem is mainly written in plain JS, based on web components (also named custom elements in the W3C specification of HTML5.3 [17]). RITS toolbox depends in several libraries including Riot.js (component management), Dygraph (visualization), domtoimage (visualization), htmlDocx (reporting), and moment.js (time data parsing). Secure access and manipulation of local files is provided by the Electron framework which encapsulates the web application into a desktop executable using the core functions of the Chromium rendering engine.

Moreover, the UI subsystem is completely written into a component-oriented programming (COP) framework. This programming methodology is standard for user interfaces and ensures some of the main OOP’s features, such as encapsulation, but it prioritizes composition over the inheritance as prevalent mechanism for reusing pieces of code.

**Fig. 2:**
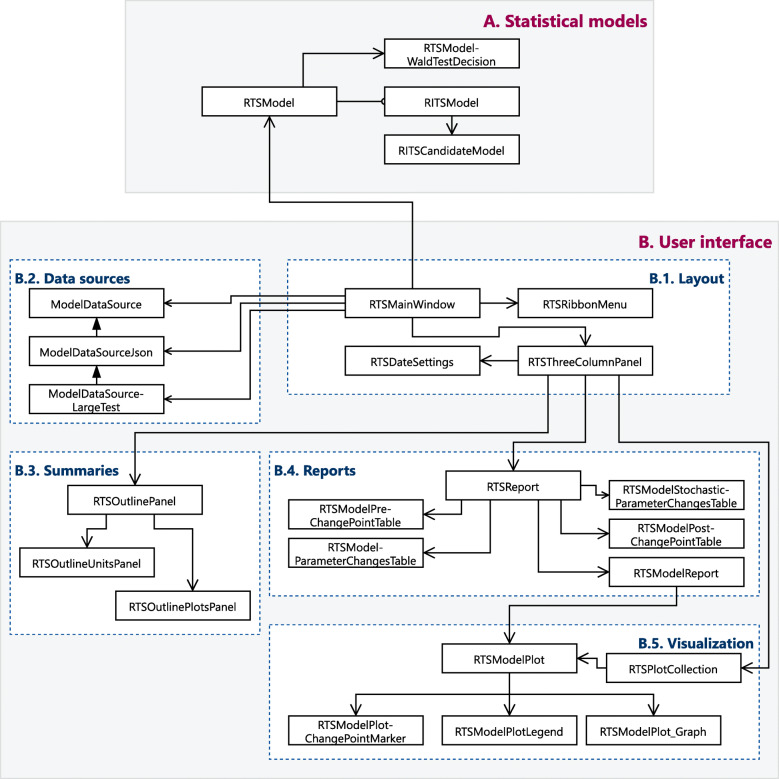
Abbreviated class diagram of the RITS toolbox with their respective subsystems: a) statistical model and b) user interface. Component packages part of the user interface subsystem are also marked with blue dotted boxes: b.1) layout controllers, b.2) data sources’ management, b.3) summary panels’ controllers, b.4) report panels’ controllers, and b.5) visualization components

**Fig. 3:**
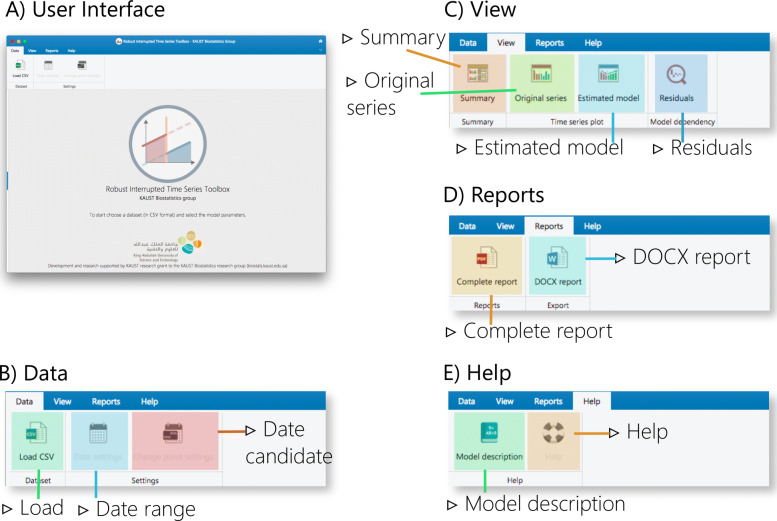
Overall user interface (a) of the RITS toolbox along with the menu options distributed in four main tabs: b) Data, c) Plots, d) Reports, and e) Help with their respective buttons

Given the complexity and the need for the user interfaces’ extensibility, the UI subsystem is grouped into five packages according to their core functionality: data source management, layout, summary panels, report panels, and visualization components. Figure 2 displays a simplified class diagram of the toolbox, where the association between the different packages and subsystems is highlighted. The final graphical interface that the user navigates in the toolbox, shown in Fig. 3, consists of multiple modules from different packages that are seamlessly combined.

The elements specified in the layout controller package describe the application’s logical structure: menus, panels, and wide-system events. The processing of input data is abstracted through several classes within the data source package. The presentation of information is carried out in two modes (overall or detailed/comprehensive), and it is managed by a) the summary package that handles the components that expose a limited, yet succinct, information of each ITS through tables and figures; and b) the report package that includes the components that display a comprehensive list of tables or figures with all relevant results. The main difference between the previous packages consists of the report components’ ability to render and export its contents to document formats, such as DOCX file formats. It should be noted that the management of every figure in the software is handled by one component in the visualization package; and that every single plotted graph includes some visualization interactivity, image exporting functions (in PNG or DOCX formats), and updating routines that are optimized to avoid overhead in memory or speed performance.

## Discussion

### User interface

The RITS toolbox has a visual structure organized through the main menu and several panels. The menu contains four tabs: Data, Plots, Reports, and Help which centralize the access to any of the panels assigned to the primary tasks: obtain estimations, generate reports, or adjust the model’s specifications. The function of each panel is described below.

#### Button data > load

This button loads a dataset into memory. Currently, the software allows comma- or tab-separated file formats as input, CSV or TSV, respectively. Those files are read and transformed into instances of the class ModelDataSource in memory.

#### Panel date > date settings

In real conditions, the user could focus the analysis on a fragment of the full dataset closer to the formal policy intervention. This could turn into more pertinent for voluminous records. This filtering by date range can be achieved in this panel. There is an interactive graph with a smaller range selector at the bottom for picking the date intervals to focus. Only the visible part of the time series will be processed.

Every update on these parameters will be applied only after clicking the button Run the model at the bottom of the screen. Note also that the settings can be changed at any moment.

#### Panel date > change-point candidates

Robust-ITS and R-MITS require determining a collection of change-point candidates through the interval {*t*^∗^−*m*,…,*t*^∗^,…,*t*^∗^+*k*}, where *m* and *k* are positive integers. In the RITS toolbox, the user can determine this set of possible change-points using the date range selector at the bottom of this panel. Consider that the change-point candidate *τ* is assumed to be in the middle of the chosen range. Once the interval has been defined, click on Run the model to update the model.

#### Panel view > summary

This panel provides a summarized perspective of the theoretical and estimated change-points for each unit. Note that this panel is automatically opened after performing any update in the model settings.

There are two subpanels in this layout. The upper panel, summary data, exhibits a table that highlights the impact (or changes) on estimates of the model (intercept, slope, and noise) as a result of the change-point at every unit. In general, this view gives a brief perspective on the strength of a particular policy.

The second subpanel, relevant results, breakdowns the information of the previous table for each unit. A graph contrasts the estimated change-point against the formal intervention point. The table makes explicit the estimated change-point, and the parameters’ values, differences and confidence intervals before and after the change-point.

#### Panel view > original series

The time series displayed in the dataset can be examined in this table. Each chart is grouped according to its unit (which can be accessed from the list on the left side).

#### Panel view > estimated model

This panel shows the original time series and the estimated mean functions displayed simultaneously organized by units. The likelihood associated with each change-point candidate is also plotted. Note that each graphic allows various interactive display actions: zoom in, zoom out, on-hover legend, and export the chart.

#### Panel view > residuals

To allow a visual inspection of the quality of the fitted model, this panel shows the residuals of the model. Three visual components are displayed per unit: the residuals over time (for a visual inspection of heteroscedasticity), box plots, and the autocorrelation function. All components are separated for comparing pre- and post-change-point.

#### Panel report > complete report

The report panel carries the essential function of presenting a full tabular and graphical description of the study on the target dataset.

To properly evaluate the presence of a change-point, the first table displays the results of applying a supremum Wald test, as it is described in the SWT section. Benjamini-Hochberg adjusted *p*-values (and critical values) are also reported in these tables. According to those estimates’ uncertainty levels, the units’ time series are labeled as with “effective change-point” (when the *p*-values are lower than 0.05) or “no change-point”.

In a second and third table, the estimates of the intercept and slopes pre- and post-estimated change-points are exposed. In each table, the estimates, *p*-values and 95% confidence intervals are detailed.

Finally, the report also includes a set of graphs associated to the fitted models of every unit: the estimated mean functions, time plots of the residuals and their autocorrelation function.

#### Button report > DOCX report

The comprehensive report of the analysis can be exported through this button into a Microsoft Word 2017 XML (DOCX) file format. Any word processor capable of parsing MHT embedded in DOCX files can adequately render the generated report. Note that it could take a few seconds to load during the first time this button is pressed due to the large number of graphs.

#### Panel help > model description

The preprint of [13] and [14], enclosing the technical description of the models, are embedded in the software for more in-depth information about Robust ITS and R-MITS.

### Illustrative example

Alongside the toolbox, we provide four different sets of sample data. Here we present an analysis sample_4.csv to illustrate the decisions users will have to make, as well as the results produced by RITS. The dataset used here provides generated data at the month level for 4 different institutes during a time period of about 7 years. This sample data is loosely based on patient-centered ITS data collected around the implementation of Clinical Nurse Leader (CNL) integrated care delivery, a nursing model that embeds a master prepared nurse into the front lines of care [18]. The outcome is based on aggregated (summed) patient experience scores generated bi-weekly between January 1, 2010 and May 24, 2017. For the purposes of illustrating the toolbox, we refer to the outcome simply by ‘outcome’ in this section.

Prior to showing any toolbox images or results of our sample data, we provide a general guide on how to use the toolbox. The standard execution workflow inside the toolbox is:

1. Initially, download the sample CSV-formatted dataset from the toolbox homepage [19] to reproduce the results of the current section or skip to step 2.
2. **Start the RITS toolbox**: A data and privacy policy disclaimer is shown automatically (Fig. 4.A). Select Accept if you adhere to these terms and proceed with the use of the toolbox.

**Fig. 4:**
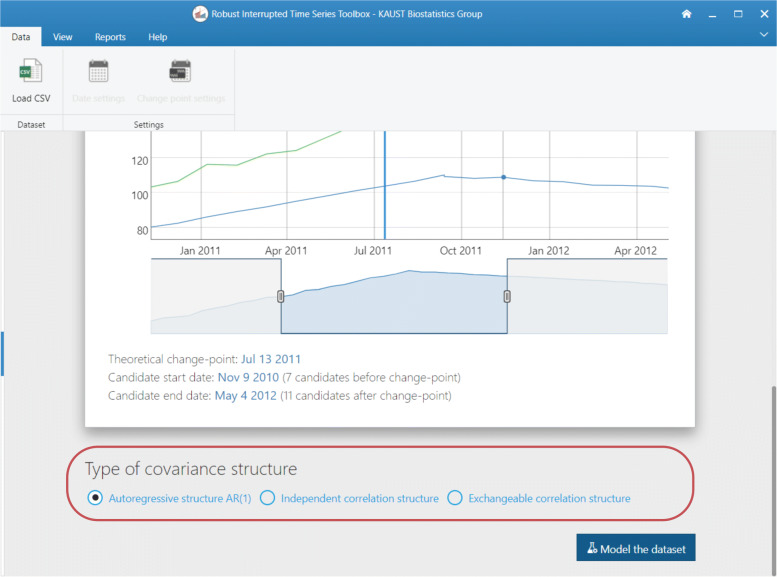
Screenshots of the RITS toolbox (I): A) data and security policy, B) loading open data file, C) defining time period analysis, D) setting change-point date candidates, E) data summary table, F) summary results per unit
3. **Load the data**: Click on the Data > Load button and select the dataset that will be used for the analyses(Fig. 4.B). If using one of the sample datasets we provide, upload the data directly/. If using your own data ensure that the data is in the correct format - refer to our sample data for the correct format. (Fig. 4.B).
4. **Define model settings**: Specify the interval of dates to be included in the analysis using the date-range selector (Fig. 4.C). Also, choose the potential change-point and formal intervention time point candidates (Fig. 4.D). Choose the appropriate correlation structure: AR(1), exchangeable or independent. Click on Run the model when both date ranges and the theoretical (or formal intervention) time point have been selected.
5. **Model summary**: RITS will automatically switch to a new view with the summary data panel (Fig. 4.E) and the relevant results panel (Fig. 4.F).
6. **Visual examination**: Click on View > Original series to check the raw time series (Fig. 5.A) or View > Estimated model to inspect the fitted mean functions overlaid with time series (Fig. 5.B). The pre- and post-estimated change-point residuals can be assessed in the residuals panel, View > Residuals, (Fig. 5.C,.D)

**Fig. 5:**
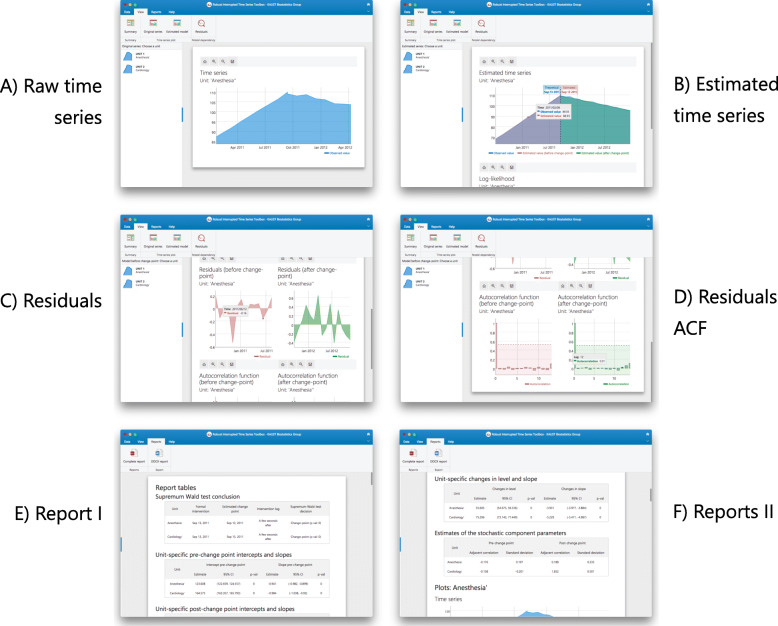
Screenshots of the RITS toolbox (II): A) raw time series plot, B) estimated model and change-point characteristics in the time series, C) residuals time-series, D) residuals auto-correlation function, E) reports I: supremum Wald test, F) reports II: intercept and slope changes pre- and post-change-point
7. **Report review**: Click on Reports > Complete report to access a comprehensive review of the results of the analysis (Fig. 5.E). Note that the first table contains the supremum Wald test. In addition, as mentioned before, this report can be exported to a DOCX by clicking on the button Reports > DOCX report (Fig. 5.F).

**Fig. 6:**
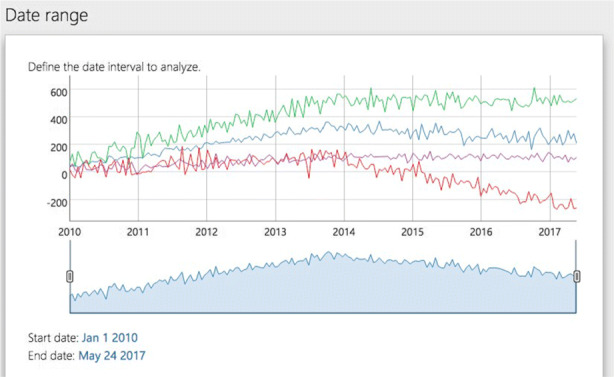
Plots the time series for each unit (or health institutes) and allows the user to select the time period of analyses. The first plot RITS provides

**Fig. 7:**
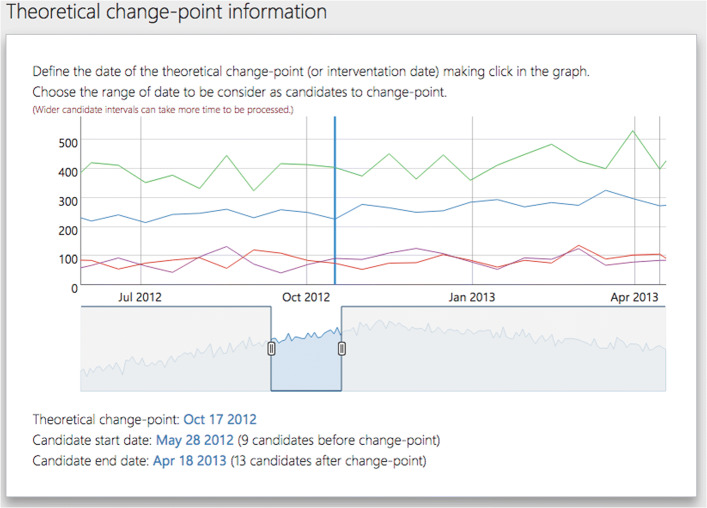
Plots and allows the user to select the set of possible change-points as well as the formal intervention or change-point, denoted as ‘theoretical’ in the plot. This is the second plot RITS produces

We followed steps 1-3 by downloading the data from the toolbox website, accepted the data and privacy disclaimer, and uploaded sample_4.csv. After uploading the data, RITS provides two plots: a plot of the time series (Fig. 6) and a plot of the set of possible change-points(Fig. 7). At first both plots span the same time period, but the user can adjust both plots as they see fit.

Figure 6 plots the time series outcome for the 4 health institutes present in our sample dataset; this is the first plot the toolbox produces. The user can choose the time period to include in the analysis with the edges of the bottom portion of this plot. We used the entire time period, and as such, did not move the edges of the bottom portion of the plot in Fig. 6.

The plot that allows the user to define the set of possible change-points and formal intervention time point (“theoretical” change-point) is provided in Fig. 7 for our specific example. We set the theoretical change-point to October 17, 2012 and the set of possible change-points to be all measurements between May 28, 2012 and April 18, 2013, for a total of 22 possible change-points. Note, if your set of possible change-points starts between May 20, 2012 and June 4, 2012 and ends between March 31, 2013 and April 18, 2013, you will get the same results we obtain in this example. This is because the measure dates occur between the selected end-point dates and not exactly on those chosen end-point dates. Figure 7 visually depicts our chosen set of possible change-points and theoretical change-point (the blue line at the top part of the figure). We chose an AR(1) correlation structure in this setting.

**Fig. 8:**
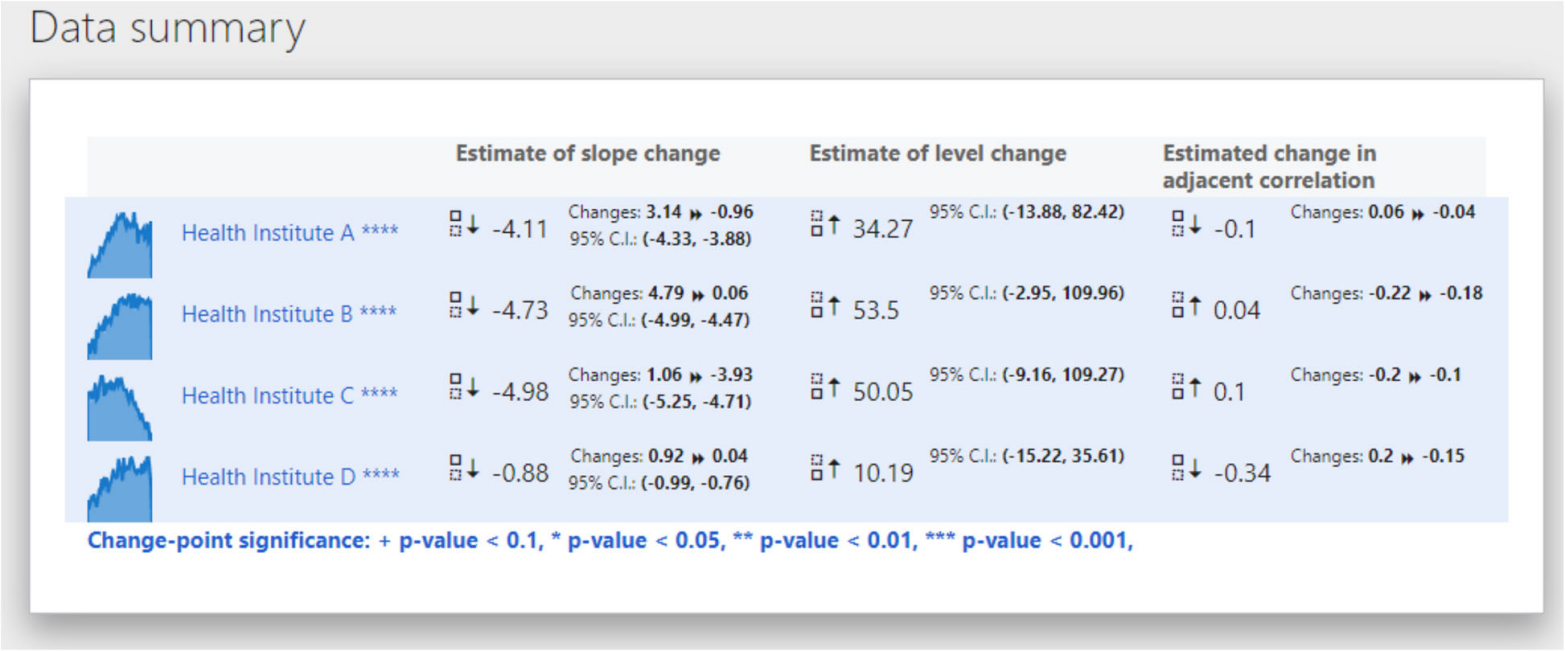
Estimates of the trend change, level change, and change in adjacent correlation for the example data

**Fig. 9:**
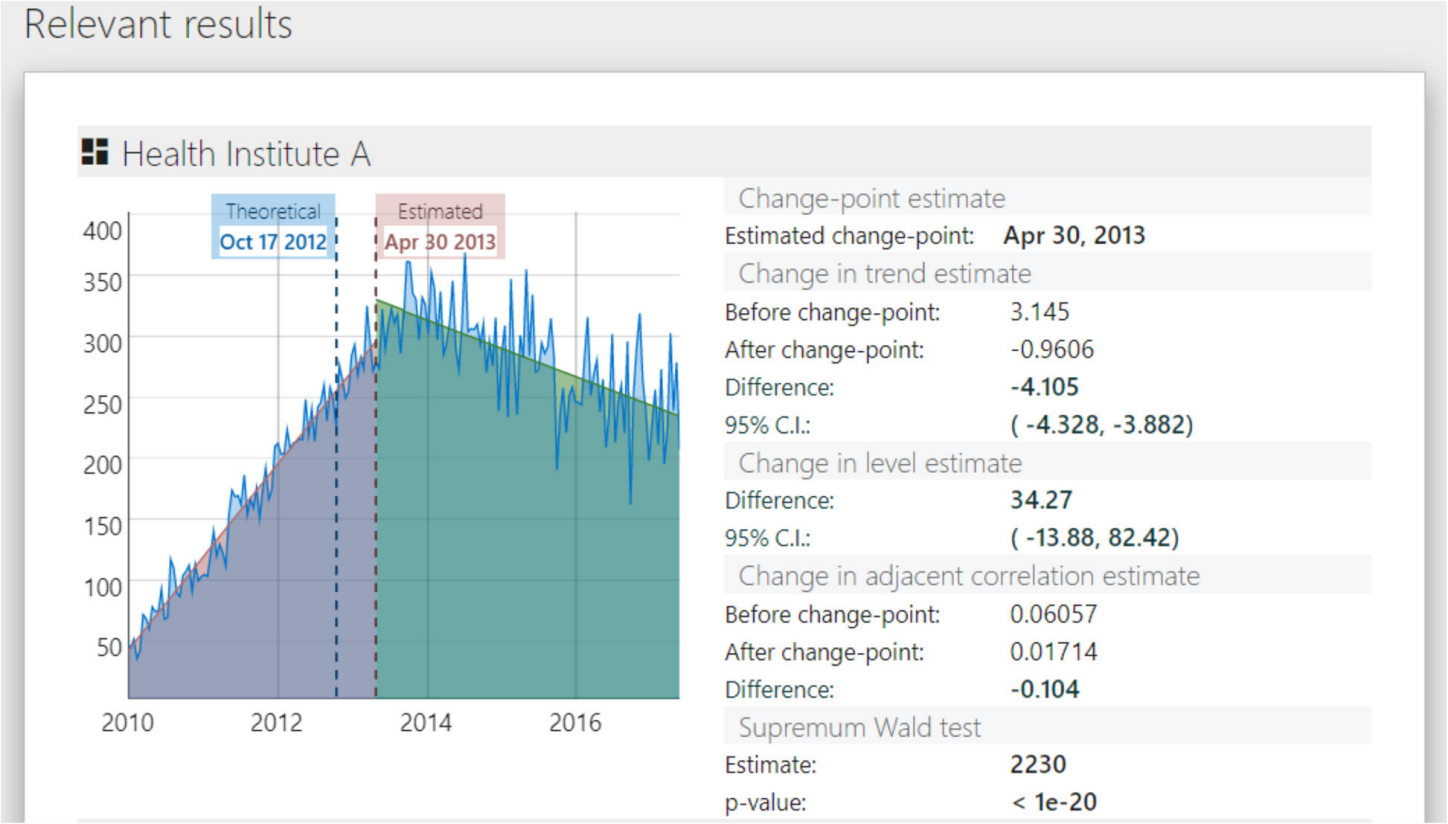
Estimates of model parameters for Health Institute A of our example data

We then clicked on Run the model and RITS switched to a new view that provided the summary data panel (Fig. 8) and relevant results panel (Fig. 9).

These two panels in Figs. 8 and 9 contain the bulk of the results. Figure 8 shows the summary data panel for all our example data and Fig. 9 includes the relevant results panel for one health institute, Health Institute A. The summary panel provides estimates of the change-point, level change, trend change, and change in adjacent correlation. From Fig. 9 we can see that for our sample data and specified set of possible change-points, the change-point is estimated to occur on April 30, 2013. The estimates of level change, trend change, and adjacent correlation change provided in Fig. 8 generally suggest that the policy intervention lead to an immediate increase followed by a gradual decrease in the outcome after the intervention.

**Fig. 10:**
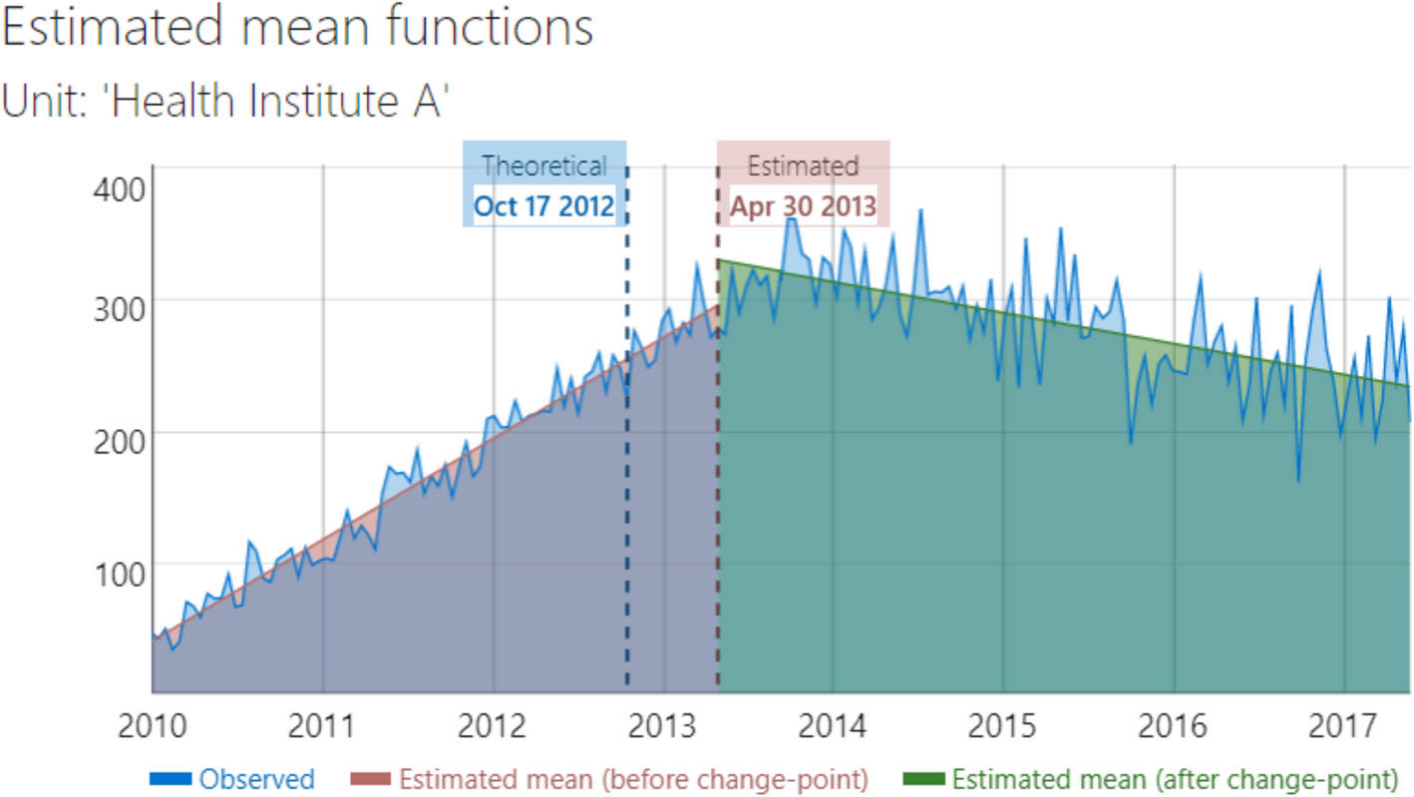
Estimated change-point and mean function for Health Institute A of our example data

Figure 10 provides the estimated mean function of Health Institute A. From Fig. 10, we see that the outcome seems to jump at the estimated change point and drops slowly after that for Health Institute A.

**Fig. 11:**
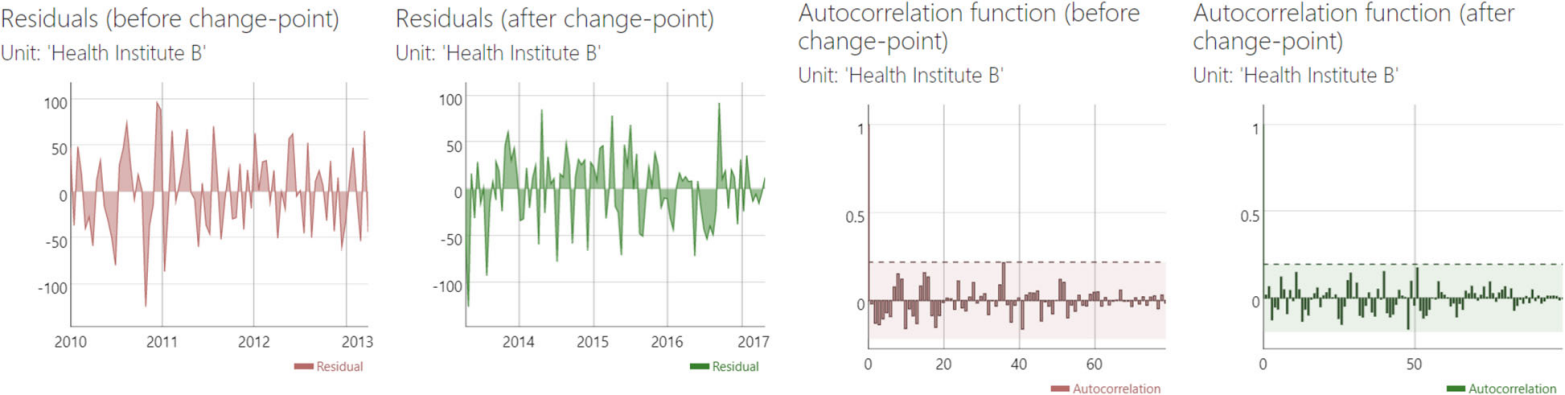
Residuals and autocorrelation function plots for Health Institute A of our example data

We then followed step 6 to view the residuals of our estimated model. The residuals, provided in Fig. 11 seem well-behaved. These there is no evidence to indicate that the model assumptions were violated.

Lastly, we followed step 7 to export our results to a word document in which all the aforementioned plots and tables are included.

### Toolbox availability

The RITS toolbox, as described above, is an open-source application available under the MIT/GPL dual license. Use the git repository [15] to find the source code of the application and their respective code documentation. Compiled binaries can be obtained from the repository page for the major operating systems (Mac OS, Windows and GNU Linux) [19].

### Existing software

The models implemented in the RITS toolbox are based on segmented regression but allow for more flexibility regarding the change-point and correlation structure. Various statistical packages and software exist for segmented regression, not for the broader Robust-ITS and R-MITS models or for a formal test for the existence of a change-point. In SAS ETS, PROC may be used to model the mean function and stochastic process without a change-point, therefore modeling time series data with no interruption [20]. PROC ARIMA, also in SAS ETS, is able to model ITS data solely with one overarching correlation structure and a pre-specified change-point or the set of possible change-points removed from the analyses [20]. Similarly in STATA, ITS data can be modeled assuming a single correlation structure for the entire observational period and with the change-point a priori specified or with the set of possible change-points removed [6]. Using the package segmentedR in R ITS data can be modeled with a fixed number of discontinuities but without consideration of the temporal correlation [21].

The models implemented in the RITS toolbox are based on segmented regression but allow for more flexibility regarding the change-point and correlation structure. Various statistical packages and software exist for segmented regression, not for the broader Robust-ITS and R-MITS models or for a formal test for the existence of a change-point. In SAS ETS, PROC may be used to model the mean function and stochastic process without a change-point, therefore modeling time series data with no interruption [20]. PROC ARIMA, also in SAS ETS, is able to model ITS data solely with one overarching correlation structure and a pre-specified change-point or the set of possible change-points removed from the analyses [20]. Similarly in STATA, ITS data can be modeled assuming a single correlation structure for the entire observational period and with the change-point a priori specified or with the set of possible change-points removed [6]. Using the package segmentedR in R ITS data can be modeled with a fixed number of discontinuities but without consideration of the temporal correlation [21].

### Limitations of existing software

Existing software limit the type of ITS analyses that researchers can conduct. Researchers are not able to model changes in both the mean functions and correlation structures, implement a test for the existence of a change-point, and estimate a change-point (if estimation is appropriate). Using existing software, researchers have to choose whether to incorporate a change in the mean functions or a change in the correlation structures. The RITS toolbox is the first piece of software that tests for the existence of a change-point, estimates the change-point (if estimation is desired), and allows for changes in both the mean functions and correlation structures at the change point. Existing software requires the user to operate a statistical programming language (e.g., STATA, SAS, and R), thus limiting user access to those with statistical programming experience. RITS does not require users to know or learn any programming language. Instead, RITS is an interactive toolbox that anyone without any statistical programming experience can use.

## Conclusion

The RITS toolbox models both single and multiple ITS for continuous outcomes, allowing for unit-specific mean functions and correlation structures, and estimating (rather than assuming) a global over-all units change-point. The mean functions and stochastic processes are allowed to vary for each unit, thus providing unit-specific estimates of the outcome’s level change, trend change, and pre- and post-intervention adjacent correlations and variances. RITS allows independent, exchangeable, or AR(1) correlation structures. See [13] or [14] for brief discussions on how to choose the correct correlation structures. We hope to incorporate more complex correlation structures, like an AR(p) with p>1 or an MA(q), into the RITS toolbox as part of our future work. For guidance on how the length of the time series and number of units impact empirical power and type one error rates please see [14].

RITS estimates an over-all-units change-point when multiple ITS are analyzed while allowing the mean functions and correlation structures to vary by unit. The change-point is estimated via a grid search over a pre-determined set of possible change-points specified by the toolbox user. When specifying the set of possible change-points, users must be cautious of competing intervention effects. Users should aim to define the set of possible change-points as the time points during which the intervention of interest plausibly impacted the outcome and exclude time periods affected by another intervention. RITS implements a formal test for the existence of a change-point over the set of possible change-points. Identification of a change-point relies upon the detection of a change in either the mean level or slope of the response post-intervention, across units.

RITS requires that the outcome is recorded at evenly-spaced time intervals. The RITS toolbox allows for missing data by linearly interpolating missing values. This imputation may lead to a small bias in parameter estimates in comparison to other methods [22, 23].We therefore recommend against analyzing series with missing data in RITS. In addition, RITS assumes that the time series are recorded for the same time points across units and analyzes data only for the overlapping time points. For example, if one time series includes monthly measures between January and December 2015 and all other units have monthly data from January 2015 to December 2016, only the 2015 data will be used. Evenly spaced measurements are needed to estimate the assumed AR(1) correlation structure properly, if assumed.

The methodology implemented in RITS assumes outcomes are normally distributed, and as such, RITS does not appropriately model discrete outcomes (e.g., does not take the mean-variance relationship into account). We plan on expanding the methodology and developing software specifically for ITS analyses of discrete responses. RITS does not allow for additional covariates (e.g., number of nurses in each unit, gender, etc.) at this time. We hope to eventually build covariates into RITS in the future. Researchers interested in adjusting for additional covariates may now utilize and extend our code to do so in their particular setting. Furthermore, when multiple time series are analyzed, RITS assumes one global overall-units change-point. This assumption may not be accurate in practice, where different units may be affected by the intervention at varying times. We will develop both methods and software for ITS data that allows for unit-specific change-points.

## Availability and requirements

- **Project name:** Robust Interrupted Time Series Toolbox
- **Project home page:**https://biostatistics-kaust.github.io/robust_time_series_toolbox/[19]
- **Operating system(s):** Mac OSX, Windows (64 bits), GNU/Linux
- **Programming language:** Nim programming language using an object-oriented programming paradigm and JavaScript
- **Other requirements:** No additional software required
- **License:** MIT/GPL dual license
- **Any restrictions to use by non-academics:** None

## Data Availability

The datasets generated and/or utilised are embedded within the toolbox and can be obtained in the ‘data-samples’ folder of the kbiostats.robust_time_series_toolbox repository [15]. All source code can also be obtained in the kbiostats.robust_time_series_toolbox repository [15].

## Abbreviations

ITS: Interrupted Time Series
RITS: Robust Interrupted Time Series
R-MITS: Robust Multiple Interrupted Time Series
SWT: Supremum Wald Test
IRLS: Iteratively Re-weighted Least Squares
OS: Operating System
OOP: Object-Oriented Paradigm
JS: JavaScript
UI: User Interface
COP: Component-Oriented Programming
CNL: Clinical Nurse Leader
